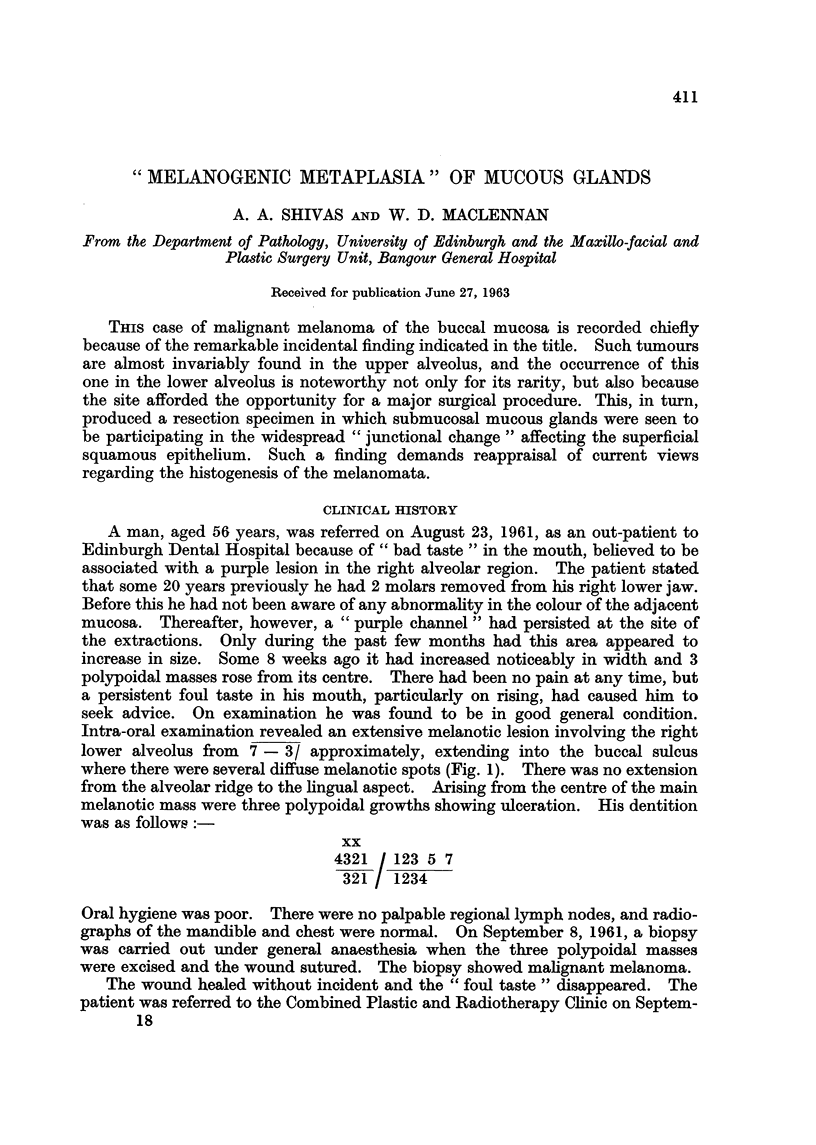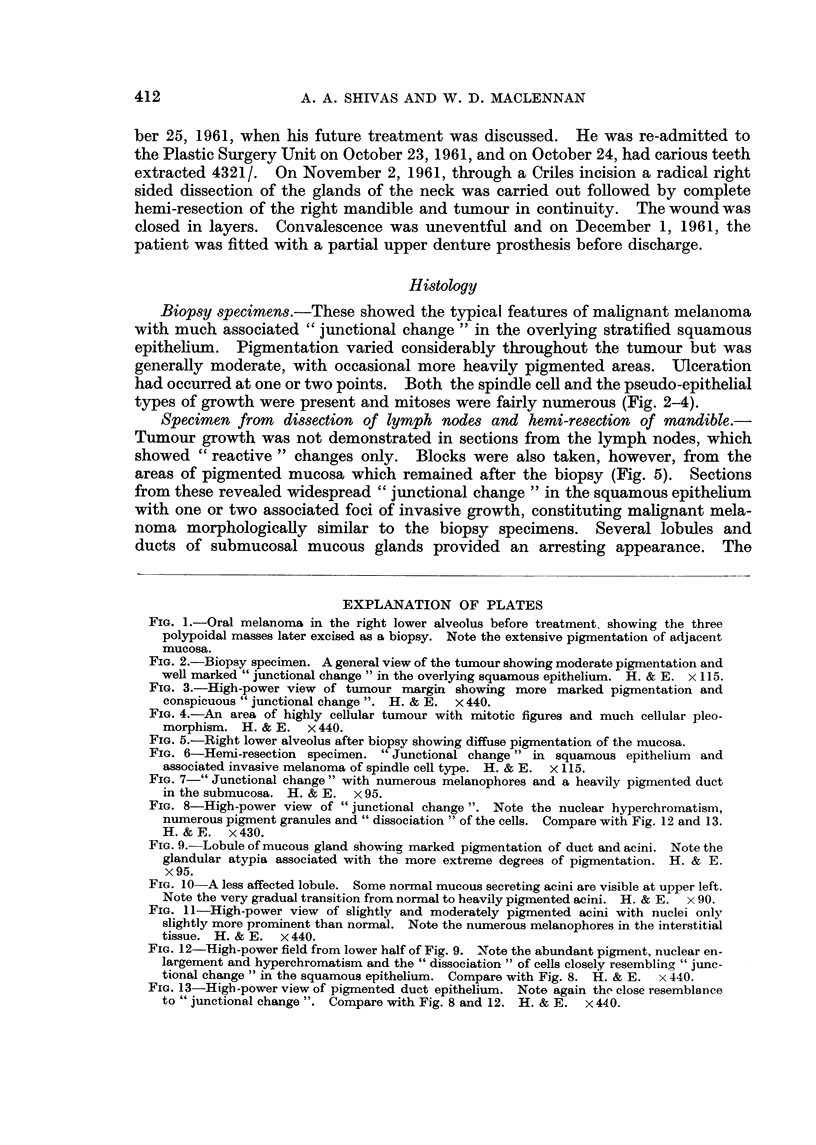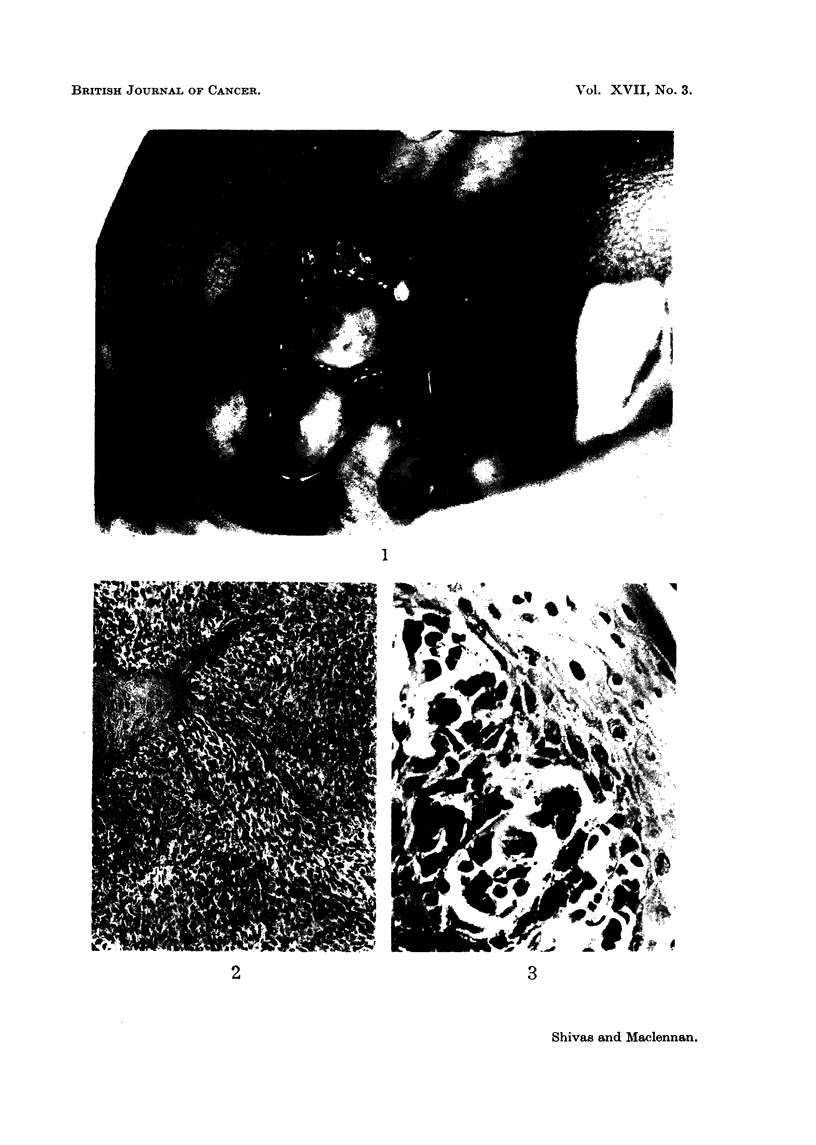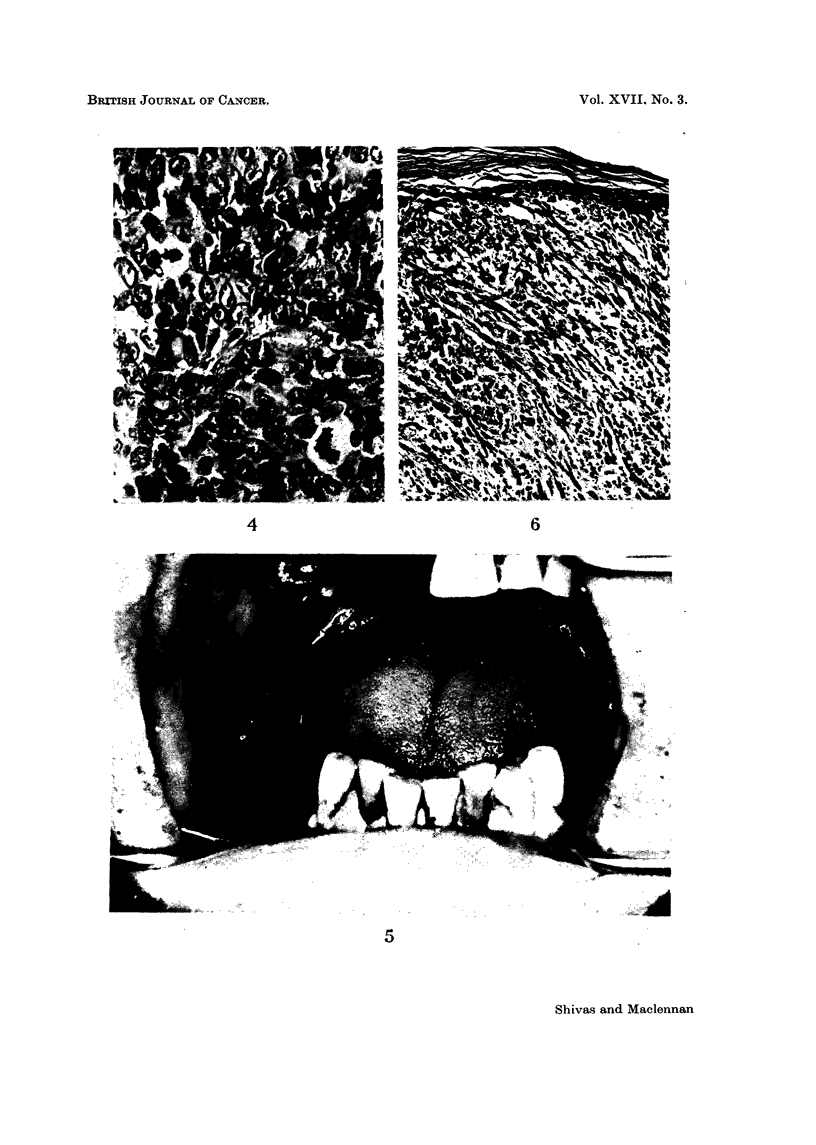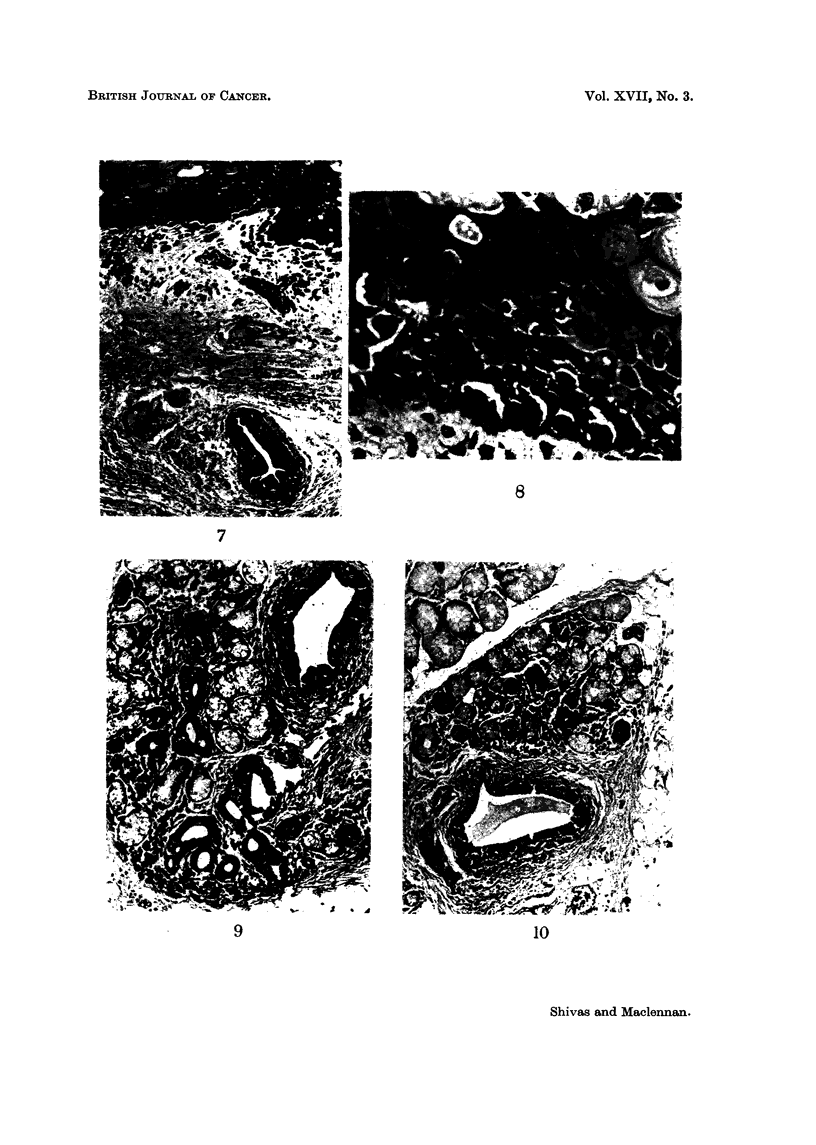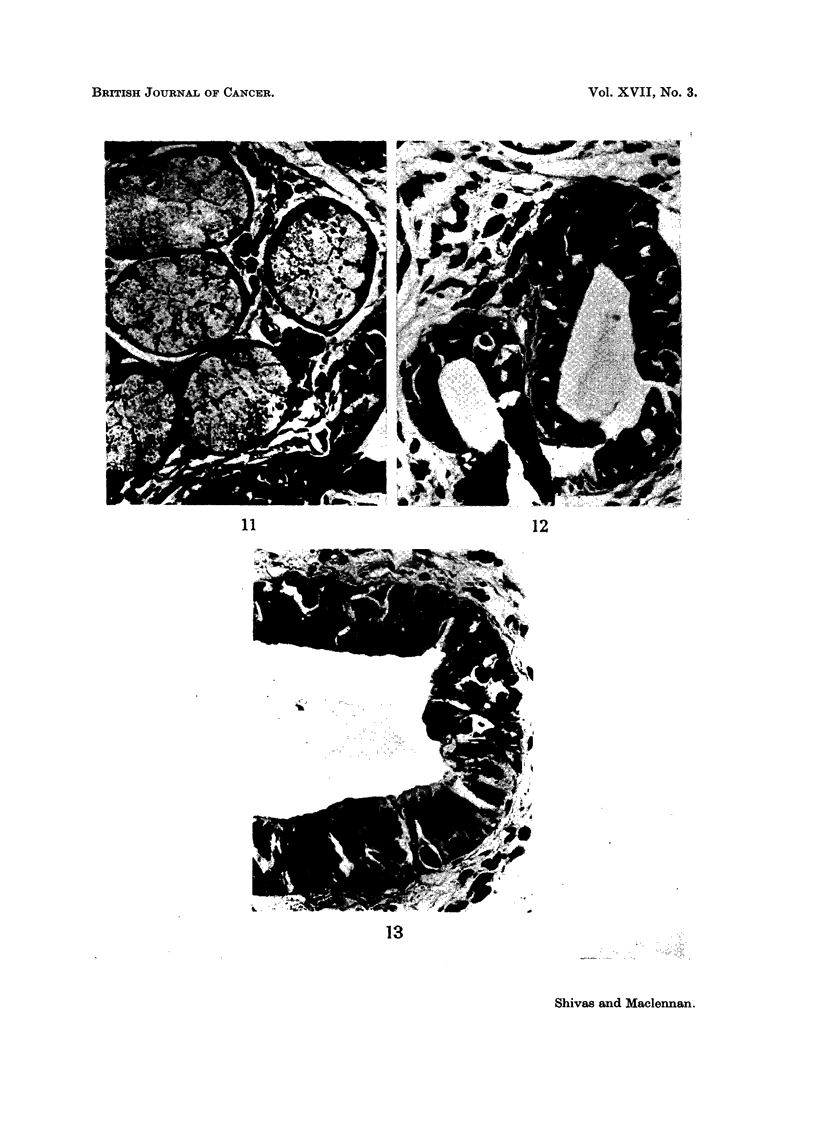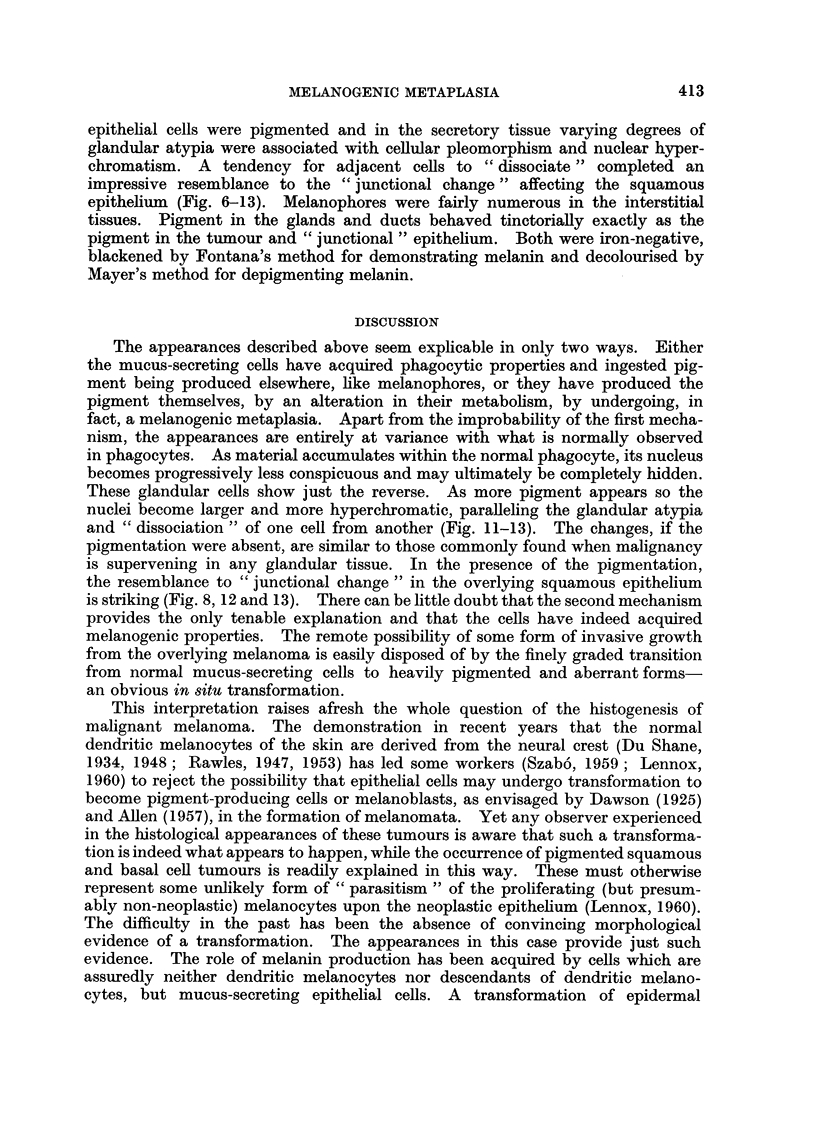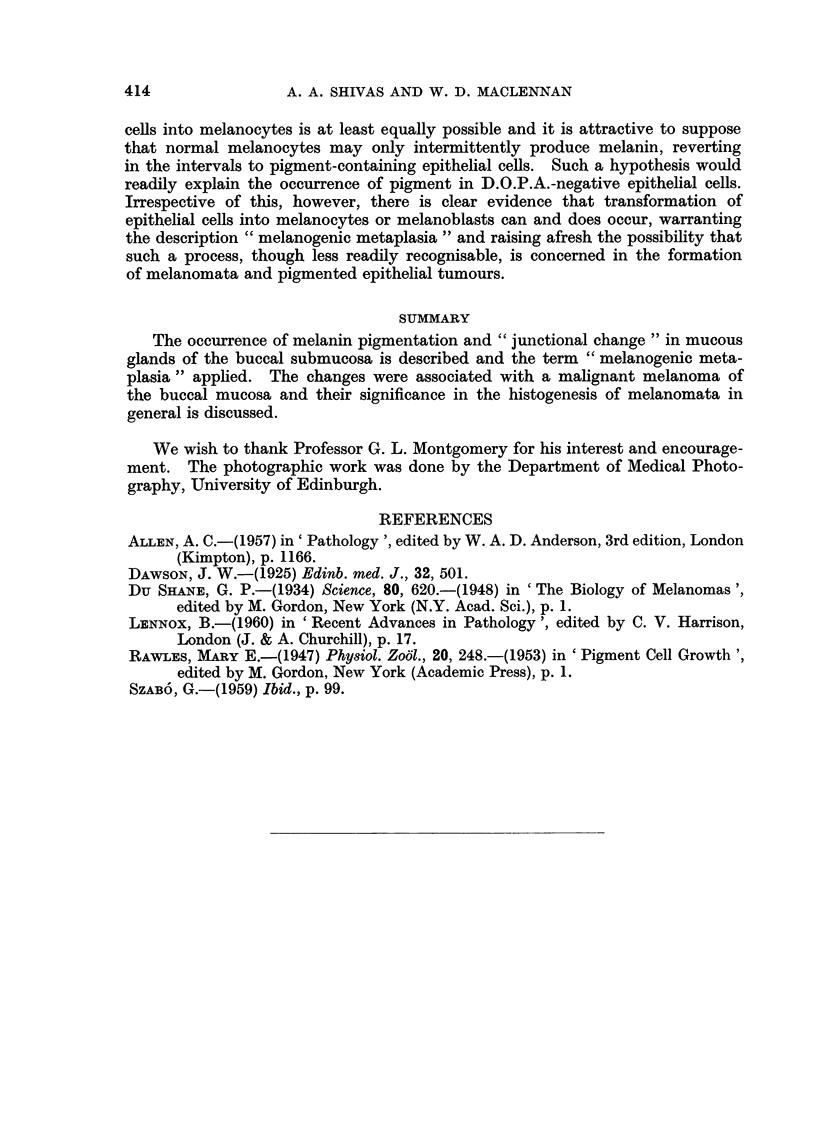# “Melanogenic Metaplasia” of Mucous Glands

**DOI:** 10.1038/bjc.1963.57

**Published:** 1963-09

**Authors:** A. A. Shivas, W. D. Maclennan

## Abstract

**Images:**


### "MELANOGENIC METAPLASIA" OF MUCOUS GLANDS

A. A. SHIVAS AND W. D. MACLENNAN

*From the Department of Pathology, University of Edinburgh and the Maxillo-facial and Plastic Surgery Unit, Bangour General Hospital*

Received for publication June 27, 1963

THIS case of malignant melanoma of the buccal mucosa is recorded chiefly because of the remarkable incidental finding indicated in the title. Such tumours are almost invariably found in the upper alveolus, and the occurrence of this one in the lower alveolus is noteworthy not only for its rarity, but also because the site afforded the opportunity for a major surgical procedure. This, in turn, produced a resection specimen in which submucosal mucous glands were seen to be participating in the widespread "junctional change" affecting the superficial squamous epithelium. Such a finding demands reappraisal of current views regarding the histogenesis of the melanomata.

### CLINICAL HISTORY

A man, aged 56 years, was referred on August 23, 1961, as an out-patient to Edinburgh Dental Hospital because of "bad taste" in the mouth, believed to be associated with a purple lesion in the right alveolar region. The patient stated that some 20 years previously he had 2 molars removed from his right lower jaw. Before this he had not been aware of any abnormality in the colour of the adjacent mucosa. Thereafter, however, a "purple channel" had persisted at the site of the extractions. Only during the past few months had this area appeared to increase in size. Some 8 weeks ago it had increased noticeably in width and 3 polypoidal masses rose from its centre. There had been no pain at any time, but a persistent foul taste in his mouth, particularly on rising, had caused him to seek advice. On examination he was found to be in good general condition. Intra-oral examination revealed an extensive melanotic lesion involving the right lower alveolus from $\overline{7 - 3}$/ approximately, extending into the buccal sulcus where there were several diffuse melanotic spots (Fig. 1). There was no extension from the alveolar ridge to the lingual aspect. Arising from the centre of the main melanotic mass were three polypoidal growths showing ulceration. His dentition was as follows :—

$$\begin{array}{r|l} \text{XX}\phantom{21} & \\ \underline{4321} & \underline{123\ \ 5\ \ 7} \\ 321 & 1234 \end{array}$$

Oral hygiene was poor. There were no palpable regional lymph nodes, and radiographs of the mandible and chest were normal. On September 8, 1961, a biopsy was carried out under general anaesthesia when the three polypoidal masses were excised and the wound sutured. The biopsy showed malignant melanoma.

The wound healed without incident and the "foul taste" disappeared. The patient was referred to the Combined Plastic and Radiotherapy Clinic on September 25, 1961, when his future treatment was discussed. He was re-admitted to the Plastic Surgery Unit on October 23, 1961, and on October 24, had carious teeth extracted 4321/. On November 2, 1961, through a Criles incision a radical right sided dissection of the glands of the neck was carried out followed by complete hemi-resection of the right mandible and tumour in continuity. The wound was closed in layers. Convalescence was uneventful and on December 1, 1961, the patient was fitted with a partial upper denture prosthesis before discharge.

### *Histology*

*Biopsy specimens*.—These showed the typical features of malignant melanoma with much associated " junctional change " in the overlying stratified squamous epithelium. Pigmentation varied considerably throughout the tumour but was generally moderate, with occasional more heavily pigmented areas. Ulceration had occurred at one or two points. Both the spindle cell and the pseudo-epithelial types of growth were present and mitoses were fairly numerous (Fig. 2–4).

*Specimen from dissection of lymph nodes and hemi-resection of mandible*.—Tumour growth was not demonstrated in sections from the lymph nodes, which showed " reactive " changes only. Blocks were also taken, however, from the areas of pigmented mucosa which remained after the biopsy (Fig. 5). Sections from these revealed widespread " junctional change " in the squamous epithelium with one or two associated foci of invasive growth, constituting malignant melanoma morphologically similar to the biopsy specimens. Several lobules and ducts of submucosal mucous glands provided an arresting appearance. The

---

EXPLANATION OF PLATES

FIG. 1.—Oral melanoma in the right lower alveolus before treatment, showing the three polypoidal masses later excised as a biopsy. Note the extensive pigmentation of adjacent mucosa.

FIG. 2.—Biopsy specimen. A general view of the tumour showing moderate pigmentation and well marked " junctional change " in the overlying squamous epithelium. H. & E. ×115.

FIG. 3.—High-power view of tumour margin showing more marked pigmentation and conspicuous " junctional change ". H. & E. ×440.

FIG. 4.—An area of highly cellular tumour with mitotic figures and much cellular pleomorphism. H. & E. ×440.

FIG. 5.—Right lower alveolus after biopsy showing diffuse pigmentation of the mucosa.

FIG. 6—Hemi-resection specimen. " Junctional change " in squamous epithelium and associated invasive melanoma of spindle cell type. H. & E. ×115.

FIG. 7—" Junctional change " with numerous melanophores and a heavily pigmented duct in the submucosa. H. & E. ×95.

FIG. 8—High-power view of " junctional change ". Note the nuclear hyperchromatism, numerous pigment granules and " dissociation " of the cells. Compare with Fig. 12 and 13. H. & E. ×430.

FIG. 9.—Lobule of mucous gland showing marked pigmentation of duct and acini. Note the glandular atypia associated with the more extreme degrees of pigmentation. H. & E. ×95.

FIG. 10—A less affected lobule. Some normal mucous secreting acini are visible at upper left. Note the very gradual transition from normal to heavily pigmented acini. H. & E. ×90.

FIG. 11—High-power view of slightly and moderately pigmented acini with nuclei only slightly more prominent than normal. Note the numerous melanophores in the interstitial tissue. H. & E. ×440.

FIG. 12—High-power field from lower half of Fig. 9. Note the abundant pigment, nuclear enlargement and hyperchromatism and the " dissociation " of cells closely resembling " junctional change " in the squamous epithelium. Compare with Fig. 8. H. & E. ×440.

FIG. 13—High-power view of pigmented duct epithelium. Note again the close resemblance to " junctional change ". Compare with Fig. 8 and 12. H. & E. ×440.

1

2

3

Shivas and Maclennan.

4

6

5

7

8

9

10

11

12

13

Shivas and Maclennan.

epithelial cells were pigmented and in the secretory tissue varying degrees of glandular atypia were associated with cellular pleomorphism and nuclear hyperchromatism. A tendency for adjacent cells to " dissociate " completed an impressive resemblance to the " junctional change " affecting the squamous epithelium (Fig. 6–13). Melanophores were fairly numerous in the interstitial tissues. Pigment in the glands and ducts behaved tinctorially exactly as the pigment in the tumour and " junctional " epithelium. Both were iron-negative, blackened by Fontana's method for demonstrating melanin and decolourised by Mayer's method for depigmenting melanin.

### DISCUSSION

The appearances described above seem explicable in only two ways. Either the mucus-secreting cells have acquired phagocytic properties and ingested pigment being produced elsewhere, like melanophores, or they have produced the pigment themselves, by an alteration in their metabolism, by undergoing, in fact, a melanogenic metaplasia. Apart from the improbability of the first mechanism, the appearances are entirely at variance with what is normally observed in phagocytes. As material accumulates within the normal phagocyte, its nucleus becomes progressively less conspicuous and may ultimately be completely hidden. These glandular cells show just the reverse. As more pigment appears so the nuclei become larger and more hyperchromatic, paralleling the glandular atypia and " dissociation " of one cell from another (Fig. 11–13). The changes, if the pigmentation were absent, are similar to those commonly found when malignancy is supervening in any glandular tissue. In the presence of the pigmentation, the resemblance to " junctional change " in the overlying squamous epithelium is striking (Fig. 8, 12 and 13). There can be little doubt that the second mechanism provides the only tenable explanation and that the cells have indeed acquired melanogenic properties. The remote possibility of some form of invasive growth from the overlying melanoma is easily disposed of by the finely graded transition from normal mucus-secreting cells to heavily pigmented and aberrant forms—an obvious *in situ* transformation.

This interpretation raises afresh the whole question of the histogenesis of malignant melanoma. The demonstration in recent years that the normal dendritic melanocytes of the skin are derived from the neural crest (Du Shane, 1934, 1948 ; Rawles, 1947, 1953) has led some workers (Szabó, 1959 ; Lennox, 1960) to reject the possibility that epithelial cells may undergo transformation to become pigment-producing cells or melanoblasts, as envisaged by Dawson (1925) and Allen (1957), in the formation of melanomata. Yet any observer experienced in the histological appearances of these tumours is aware that such a transformation is indeed what appears to happen, while the occurrence of pigmented squamous and basal cell tumours is readily explained in this way. These must otherwise represent some unlikely form of " parasitism " of the proliferating (but presumably non-neoplastic) melanocytes upon the neoplastic epithelium (Lennox, 1960). The difficulty in the past has been the absence of convincing morphological evidence of a transformation. The appearances in this case provide just such evidence. The role of melanin production has been acquired by cells which are assuredly neither dendritic melanocytes nor descendants of dendritic melanocytes, but mucus-secreting epithelial cells. A transformation of epidermal cells into melanocytes is at least equally possible and it is attractive to suppose that normal melanocytes may only intermittently produce melanin, reverting in the intervals to pigment-containing epithelial cells. Such a hypothesis would readily explain the occurrence of pigment in D.O.P.A.-negative epithelial cells. Irrespective of this, however, there is clear evidence that transformation of epithelial cells into melanocytes or melanoblasts can and does occur, warranting the description " melanogenic metaplasia " and raising afresh the possibility that such a process, though less readily recognisable, is concerned in the formation of melanomata and pigmented epithelial tumours.

### SUMMARY

The occurrence of melanin pigmentation and " junctional change " in mucous glands of the buccal submucosa is described and the term " melanogenic metaplasia " applied. The changes were associated with a malignant melanoma of the buccal mucosa and their significance in the histogenesis of melanomata in general is discussed.

We wish to thank Professor G. L. Montgomery for his interest and encouragement. The photographic work was done by the Department of Medical Photography, University of Edinburgh.

### REFERENCES

ALLEN, A. C.—(1957) in ' Pathology ', edited by W. A. D. Anderson, 3rd edition, London (Kimpton), p. 1166.
DAWSON, J. W.—(1925) *Edinb. med. J.*, **32**, 501.
DU SHANE, G. P.—(1934) *Science*, **80**, 620.—(1948) in ' The Biology of Melanomas ', edited by M. Gordon, New York (N.Y. Acad. Sci.), p. 1.
LENNOX, B.—(1960) in ' Recent Advances in Pathology ', edited by C. V. Harrison, London (J. & A. Churchill), p. 17.
RAWLES, MARY E.—(1947) *Physiol. Zoöl.*, **20**, 248.—(1953) in ' Pigment Cell Growth ', edited by M. Gordon, New York (Academic Press), p. 1.
SZABÓ, G.—(1959) *Ibid.*, p. 99.